# Adaptive Decision Method in C3I System

**DOI:** 10.1155/2022/6967223

**Published:** 2022-08-19

**Authors:** Kun Gao, Hao Wang, Joanicjusz Nazarko, Marta Jarocka

**Affiliations:** ^1^Zhejiang Business Technology Institute, Ningbo 315012, China; ^2^NingboTech University, Ningbo 315012, China; ^3^Bialystok University of Technology, Bialystok 15-351, Poland; ^4^Bialystok University of Technology, Faculty of Engineering Management, Bialystok, Poland

## Abstract

As an important system in the national defense and military information construction, the command, control, communication, and intelligence (C3I) system urgently needs to establish an adaptive process to deal with the dynamic operating environment and changeable task requirements to ensure the long-term effective and stable operation of the system. As an important part of this process, the adaptive decision method should have the ability of online trade-off decision. Therefore, this paper presents an adaptive decision method based on parallel computing and optimization theory. This method combines operational requirements and commander preference to achieve the parallel adaptive decision solution. The experimental results show that the presented decision method can generate online trade-off strategies to deal with typical command and control scenarios of damage replacement in a simulated environment, effectively guide the system to carry out adjustment behavior, and achieve the goal of dynamic response to environmental changes and task changes.

## 1. Introduction

The command, control, communication, and intelligence system plays a key role in national defense and military information construction. By comprehensively collecting and analyzing situation information and dynamically managing and allocating operational resources, such systems provide commanders with the ability to quickly integrate human, physical, information, and other resources to assist commanders in implementing the most appropriate battlefield decisions. At present, the operation environment of the C3I system is prone to environmental changes such as adding new computing nodes, key transmission link failure, efficiency degradation, and cyber-attack. Moreover, the task requirements faced by the C3I system are dynamic and phased, and unexpected task change requirements will occur at any time during the operation of the system. These changes in the operating environment and the task change requirements, if not responded in a timely and reasonable manner, may lead to the overall failure of the system in serious cases. Therefore, the C3I system urgently needs to have an adaptive ability so that the system can adapt to the dynamically changing environment and combat tasks by adjusting its own organizational structure or behavior.

To realize this adaptive capability, the US army put forward the “OODA” (observe-orient-decide-act) theory and established an adaptive process including “observation, judgment, decision, and action” to realize the real-time response of the system to environmental changes and task changes. Among them, the observation link is to perceive or observe the system operation environment or demand changes, and collect information and data from them. The judgment phase is to analyze and evaluate the current system status and process relevant information and data. The decision process is to formulate adjustment strategies and select appropriate strategies according to the operating environment information and the current system status. The action phase is to adjust the system according to the selected strategy. It can be seen that the adjustment strategy generated in the decision process ultimately determines how the system adapts. This link is the key to determine whether the C3I system can be successfully adjusted to adapt to the current environment or change the task. There are two urgent capacity needs in the decision link in the command and control field.

Firstly, because the C3I system operates in a highly dynamic environment, environmental changes occur frequently and there may be multiple environmental changes at the same time. Considering that the C3I system itself is a physical system with a complex operation mechanism, according to the laws of the real world and practical experience, changes at the same time may have a certain correlation. This association will lead to potential conflicts in the adjustment strategies of each change [1]. For example, in response to multiple changes occurring at the same time, the system will adopt multiple adjustment strategies to adjust the same operational resource in different ways. To solve the above problems, the adaptive decision method is required to be able to weigh the changes in conflict relationships and produce a compromise.

Secondly, because the system operating environment is changing rapidly and the command system's internal state and behavior space are exploding, it is almost impossible to enumerate all possible adjustment strategies and predict the actual effectiveness of the strategies before the system runs. The uncertainty of this strategy and the unpredictability of its effect lead to that the adaptive decision method can not correctly deal with the known changes only by the predetermined strategy, and cannot deal with the unknown changes without relevant strategies. Therefore, the adaptive decision method must have the online decision ability to dynamically generate adjustment strategies according to the changing situation.

However, the existing C3I system cannot fully meet the above two capability requirements. For example, the rule/policy-based method needs to predefine adaptive policies before the system runs, which cannot support online running. The decision method based on objective, utility function, and optimization function can only deal with a single change each time, which is easy to produce conflicting adaptive strategies. Although there are some studies that generate compromise strategies by defining the weight relationship between changes, the priority relationship between changes will evolve during the operation of the system, such studies cannot ensure that it is always effective [2]. However, the adaptive decision method of general software is not applicable when it is applied in the field of command and control. For example, although adaptive decision methods based on Markov chain and Bayesian network have the ability to deal with changing uncertainties, such methods need to establish a system state transition model in advance, which is more complex for the C3I system. The decision methods based on reinforcement learning need to repeatedly try errors according to the environmental feedback to produce the optimal strategy, which is not suitable for the application field of C3I system with high-security requirements.

This paper intends to adopt the search-based software engineering (SBSE) theory, regard the adaptive decision as the search optimization problem of evaluating and selecting the optimal strategy in the search space composed of adjustment strategies, and adopt the search optimization method to establish an adaptive decision method that meets the needs of online trade-offs to effectively respond to environmental changes and task demand changes. This kind of decision method can adopt the multi-objective search optimization technology to realize the trade-off processing of multiple changes without defining the change priority and can dynamically generate the corresponding adjustment strategy based on the online search of unexpected changes.

However, when applying this method to solve the adaptive decision problem of C3I system, there are still practical problems such as efficiency improvement and strategy selection. Firstly, the computational efficiency of this method is affected by the size of the search space. The C3I system requires a high real-time performance of the adaptive process. Therefore, it is necessary to ensure that this method can produce decision results quickly. Secondly, when this kind of method makes trade-off decisions for multiple objectives, it will produce an optimal policy set, that is, the frontier. The strategies within the frontier can no longer distinguish the advantages and disadvantages according to the optimization objectives. However, the C3I system must accurately adjust the system structure, behavior, or parameters according to the only adjustment strategy. Therefore, it must be ensured that this method can select the most applicable strategy from the frontier.

This paper proposes a self-adaptive decision based on parallel retrieval optimization for the C3I system. Though transforming the adaptive decision problem into a search optimization problem, a multi-objective optimization algorithm is designed to meet the needs of the C3I system for online decision and decision trade-off. At the same time, a strategy selection method based on post optimization theory is designed to improve the decision efficiency, which can select the most applicable strategy at the moment.

The organizational structure of this paper is as follows:

Section 1 of this paper introduces the relevant background knowledge.  Section 2 gives the research framework of this paper and explains the relationship between the research work.  Section 3 introduces the core research work of this paper, namely, adaptive decision problem modeling, adaptive decision method based on parallel particle swarm optimization and genetic algorithm (PSOGA), and multi-index ranking method based on Elimination et Choice Translating Reality (ELECTRE). In Section 4, the typical adaptive scenarios and large-scale simulation scenarios of the C3I system are used to compare the proposed methods and related research methods, and the experimental results are analyzed and discussed.  Section 5 summarizes the work of this paper.

## 2. Related Works

This paper mainly analyzes the current research status of mainstream adaptive decision methods and their application in the C3I system and focuses on the comparative analysis between this work and the existing search-based adaptive decision methods.

### 2.1. Adaptive Decision Method and Its Application in Command and Control Field

Reference [3] divides the current mainstream decision methods for general fields into four types based on rules/strategies, models, utility functions, and objectives according to the knowledge types adopted by adaptive decision methods.

The adaptive decision method based on rule/policy obtains the appropriate adaptive strategy through rule matching or rule reasoning. Due to the high processing efficiency of this method [4], this method has been widely used in the C3I system and has played an important role in the C3I system until now. Reference [5] put forward a rule-based dynamic loading method of functional modules to realize flexible reconfiguration of C3I system with diversified requirements. However, this kind of method has a fatal defect, that is, it needs to define all candidate strategies statically and determine the mapping relationship between adjustment schemes and events in advance. To solve this problem, there are many ideas for improvement at home and abroad, mainly through online modification and refined adjustment of the strategy itself.

The goal-based adaptive method takes the system goal as the decision goal and takes the satisfaction degree of the strategy to the goal as the measurement standard to compare the advantages and disadvantages of different strategies to select the adaptive strategy that best meets the requirements of the system goal. Related research work can be divided into two categories. One is to establish an adaptive demand model and establish an adaptive strategy based on it [6]. The other is to judge and select the optimal adaptive strategy by measuring the satisfaction of the existing adaptive strategy with the goal [7]. In the field of command and control, there are some relevant research works carried out by using ant colony algorithm and and/or tree search method, but most methods still only consider dealing with single objective problems or multi-objective problems through fixed priority.

Utility function refers to the relationship function between the goal completion (i.e., revenue) and the cost (i.e., cost) caused by adaptive adjustment. The utility function-based adaptive method takes system utility as a decision objective, uses the utility function to measure the system utility that can be increased after different adaptive strategies adjust the system to select the optimal adaptive strategy [8]. Similar to the goal-based method, in the field of command and control, most researchers use PCA, AHP, or FCE to calculate the experts' scores on utility, and then select the optimal strategy. This process still needs to rely on a lot of expert experience, and cannot effectively deal with unknown changes online.

The model-based method itself can be divided into model-related method and a model-free method according to the relevance to the model. Based on the model method, the system state trend can be predicted and analyzed by establishing the model and obtaining the system state information. The commonly used models include deep neural networks [9], reinforcement learning [10], etc. In recent years, the emerging adaptive decision methods based on the Markov decision process [11] and Bayesian network [12] are also model-based decision methods in essence. This kind of method needs to establish a complete system model and constantly try and error, which costs a lot. At present, it has not been widely used in the field of command and control.

### 2.2. Search-Based Adaptive Decision Method

At present, search-based software engineering has become a research hotspots. However, the research on the application of this theory to adaptive software is still in the exploratory stage [13], and the main work focuses on the framework design [14], architecture optimization [15], software testing [16], development process and workload estimation [17], programming and repair [18] of adaptive software systems. For example, the Moses framework is an adaptive system optimization framework for service-oriented computing [19], and the research on the combination of adaptive software architecture and search-based software engineering proposed by Ref. [20]. At present, there is little research on the combination of adaptive decision and search-based software engineering.

The most relevant research work of this paper is proposed by Ref. [21]. Considering the relationship between software changes and dynamic priority changes, this method uses evolutionary programming combined with basic adaptive strategies to generate policies. However, the overall process of this method adopts the static off-line operation mode [22], which cannot handle the decision problems with strong real-time performance. The decision method proposed in this paper can dynamically form the scheme space and realize online decisions according to real-time software changes, which can be better applied to adaptive software systems with greater dynamics and uncertainty.

## 3. Adaptive Decision Method Based on Parallel Search Optimization

### 3.1. Adaptive Decision Problem Modeling

According to the above basic theory of mapping adaptive problems to optimization problems, this section describes its specific methods. In the adaptive problem, the variable that affects the adjustment result is the adaptive strategy. Therefore, this paper takes the adaptive strategy as the decision variable of the adaptive problem. In the C3I system, the adaptive strategy contains a large amount of system information and system behavior. This paper defines the components of the adaptive strategy as variable points (VP), that is, the adjustable objects that can affect the adjustment results in the adaptive adjustment process, These adjustable objects not only include the C3I system resources (such as CPU and memory utilization of computing nodes), the organizational structure of the C3I system (such as the number of combat units, deployment location.), the behavior or parameters of the component units (such as the functions of the system component units.) but also include the adjustable attributes (such as network bandwidth.) during the operation of the C3I system.

Due to the different values of variable points, variable points can be divided into discrete variable points, continuous variable points, and quantitative variable points.

- Discrete variable point means that the variable value is a discrete value.
- Continuous variable point means that the value of variable point is continuous.
- Quantizing variable points means that some of the variable points do not have specific digital values, so they need to be quantized.

**Figure 1:**
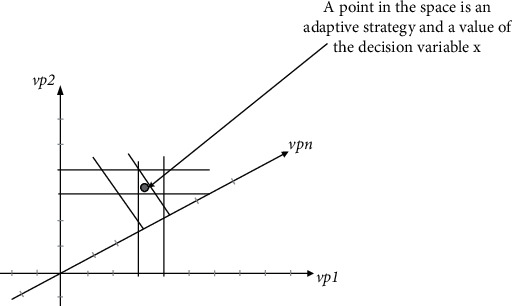
Adaptive decision problem modeling.

Since decision variables refer to adaptive strategies, the combination of these variable points forms an adaptive strategy set, that is, the value of decision variables, which is also called solution space in search-based software engineering. As shown in Figure 1, the solution space formed by the combination of decision variables is shown, in which each variable point is a coordinate axis in the solution space, and a point in the solution space represents a combination of variable points.

The solution space established in the above way is generated through online analysis of variable points (i. e., system state and behavior) and there is no predetermined relationship. At the same time, since each point in the solution space is independent, there is no conflict between different strategies. This method can effectively avoid policy conflict and dynamically select the optimal adaptive policy to realize online decisions.

#### 3.1.1. Objective Function

The optimization goal of the adaptive decision in the C3I system is to make the system that needs adaptive adjustment adapt to software changes to achieve the desired system goal. In this paper, software change specifically refers to the internal changes of the system caused by environmental changes and changes in battlefield requirements. The system objective which is expected to be stable after adjustment is taken as the objective function and it is divided into two parts: direct correlation function and indirect correlation function.

First of all, it is necessary to define the desired system objectives at the later stage of software change. System objectives can be obtained in two ways. One is to acquire the experience of experts in the field of accusation, and the other is to conduct multiple experiments by simulating battlefield environment changes and demand changes many times to observe the affected system resources and behaviors. The affected part is the objective function directly related to this change. For example, in the “load balancing” scenario, the “node load overload” change may occur. To obtain the direct objective function of this change, it is necessary to repeat the experiment. By analyzing the system operation log, it can be found that the “system response time” is affected. Therefore, “response time” is a directly related objective function of this change.

Second, there may be a link between environmental changes and needs. Therefore, the directly related objective function cannot be simply considered. Through the analysis of various software changes, the relevant variable point information can be defined. The system objective affected by the value change of these variable points is called the indirectly related objective function of the change. By introducing the indirect objective function into the adaptive decision problem, a series of system oscillations, such as repeated adjustment, caused by the relationship between software changes can be avoided to a certain extent. For example, in the “load balancing” scenario, the variable point related to the change of “node load overload” is the task allocation of the node, and this variable point affects the running cost of the system. Although the system operation cost is not affected by “node load overload” for the time being, in the long run, incorporating it into the indirect objective function can avoid software changes such as “excessive system operation cost” caused by improper adjustments.

Due to the complex operating environment of the C3I system, there may be multiple environmental changes and task requirements changes at the same time. An adaptive decision needs to consider multiple changes at the same time. Therefore, it is necessary to combine and de-duplicate the direct and indirect correlation objective functions of these changes as the overall objective function of adaptive decision. There is no need to define any priority relationship between these objective functions, which will be defined in the form of quantitative formula. The specific formula content needs to be established according to the specific decision needs of different systems.

#### 3.1.2. Constraint Function

In the adaptive decision problem, the constraint function is still an equality or inequality constraint form in the traditional optimization problem, which is mainly used to limit the value range of variable points, but its sources include the following two kinds.

- The variable point constraint mainly comes from the special requirements of adaptive adjustment system runtime context on the value of variable points.
- Functional constraints are derived from users' functional expectations of the system. The adaptive strategy should not only ensure the optimization of the objective function in the decision problem but also ensure that after the system is adjusted based on the strategy, the service that the system must provide, and the service level cannot be affected. In other words, this kind of constraint function is mainly used to ensure that the value of the variable point will not affect the necessary external functions provided by the system for users.

### 3.2. Adaptive Decision Method Based on Parallel PSOGA

According to the method described in the previous section, this paper realizes the modeling of adaptive decision problem under the C3I system. How to select the optimal adaptive strategy from the feasible solution space determined by the model, that is, the feasible adaptive strategy space, is the problem to be solved in this section.

At present, the genetic algorithm and the swarm intelligence algorithm are the most widely used multi-objective optimization algorithms in the industry [23]. Genetic algorithm has the advantages of global search, discrete continuous space, global movement, and extensive search, but it also has the problems of slow convergence, falling into local optimization, and is premature. The swarm intelligence algorithm is suitable for real scenes because of its simple structure design, but it also has the same premature problem as genetic algorithm, low search accuracy, and slow iteration speed. However, the adaptive decision problem of the C3I system has strong real-time performance, and its decision efficiency is the first element to be guaranteed. If the decision results cannot be produced quickly, the system and operating environment are likely to continue to change in the decision process, and then the selected adjustment scheme is likely to be no longer suitable for the new environment.

The proposed algorithm is mainly composed of two parts, one is the multi-objective optimization algorithm NSGA-II used for the early iteration of the algorithm and the other is the NSGA-II algorithm used for the later accurate search of the algorithm. NSGA-II algorithm has a small time cost, so it is used to realize the comprehensive coverage search of the policy space in the early stage of the algorithm. Since the points in the feasible adaptive strategy space are distributed discretely, the discrete MOPSO algorithm is used to search the local elite population in depth in the late stage of the algorithm implementation to shorten the convergence time of the algorithm. The algorithm flow is shown in Algorithm 1.

As shown in Algorithm 1, the process of parallel PSOGA algorithm is as follows.

1. Initialization. In this step, the group characteristics required for the implementation of NSGA-II algorithm and MOPSO algorithm are determined. It mainly includes population size, dimensions, value constraints on each dimension, initialization information of particles, and value constraints on the position and speed of particles in each dimension.
2. Subpopulation evolution. Nondominated sorting, congestion calculation, crossover, and mutation operations are performed on individuals to update the fitness value of individuals. Different subpopulations exchange dominant individuals through migration operators and evolve iteratively.
3. Depth optimization. After reaching the termination conditions of NSGA-II, determine the population size, dimensions, values in each dimension, and the initial position and initial velocity of particles. The upper and lower limit values of the position and velocity of particles in each dimension are the initial particle swarm along the strategy, and the redundant particles are discarded according to the fitness ranking and congestion ranking results. Calculate the fitness value of each particle, perform nondominated sorting, calculate and record the optimal position ITP of each individual and the optimal position GTP of the population, save GTP in the optimal set (archive), select a unique GTP, and then update the speed of each particle.
4. Algorithm termination. Judge the number of iterations and decide whether to end the algorithm. If the algorithm termination conditions are met, the optimal set will be nondominated sorted and the leading edge will be the output.

In combination with the above algorithm flow, the design details of the PSOGA algorithm in this paper are given, as shown in Table 1. The checkmark indicates that the corresponding algorithm needs to consider the design content.

There are some challenges and difficulties in the design of a parallel PSOGA algorithm. Firstly, because genetic algorithm generally uses the binary coding method to encode individuals, this coding method is easy to implement evolutionary operations such as mutation. However, the value of decision variables in the adaptive decision problem is discretized. If binary coding is used, it is easy to produce invalid values, and because the value of decision variables may change at any time, the coding method for adaptive decision variables must support the flexible adjustment of the value of decision variables. Secondly, in the early iteration of the algorithm, this paper will segment several sub-populations to realize parallel evolution. However, if the population segmentation is too large, the convergence efficiency will be too low, and if the population segmentation is too small, it will lead to a local optimal solution. It is necessary to consider finding a balance between the result quality and the convergence speed and provide better results within an acceptable time range as far as possible.

#### 3.2.1. Coding Method

**Figure 2:**
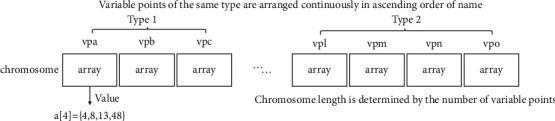
Array-based chromosome coding process.

In the running system, the value of decision variables is more likely to change. Therefore, adaptive decision variables need to support the flexible adjustment of variable values at the coding level. In particular, this paper proposes an array coding method to code the decision variables, namely the individuals in GA and PSO algorithms. As shown in Figure 2, all values of each variable point in the decision variable are mapped to an array. Each item of the array corresponds to a value. The coding length of decision variables depends on the number of variable points in the practical problem of adaptive decision.

#### 3.2.2. Group Optimal Selection

For MOPSO, this paper uses the technique for order preference by similarity to an ideal solution (TOPSIS) to select the optimal population. In the first iteration, the optimal extremum and the worst extremum of each objective function will be pre-selected as the ideal optimal value and the ideal worst value. By calculating the distance between each individual optimal value and the ideal optimal value and the ideal worst value, the individual optimal value closest to the ideal optimal value and farthest from the ideal worst value is the group optimal value. In addition, by comparing the nondominated ranking relationship between the latest optimal value and the historical group optimal value, the latest group optimal value is determined. This method can effectively avoid the influence caused by the inaccuracy of random selection methods and the computational complexity of aggregation density methods.

#### 3.2.3. Inertia Weight Design

For the inertia weight, the calculation process is shown in

(1) $$\begin{array}{c} w=W_{ub}-\frac{\text{times}}{\text{MAX}\_\text{ITER}}\left(W_{ub}-W_{lb}\right), \end{array}$$

where, times represents the current iteration times, wub and WLB represent the upper and lower bounds of the inertia factor respectively. In this paper, it is specified that wub is taken as 1 and WLB is taken as 0. MAX_ ITER represents the maximum iteration period. The inertia weight in formula (1) will decrease as the number of iterations increases.

#### 3.2.4. Termination Condition Design

Considering that the adaptive decision problem needs to obtain the decision results as soon as possible, and the general practical engineering problems are not particularly required to obtain the optimal solution in the real sense, this paper uses the specified number of iterations to terminate the algorithm when it reaches.

The value of the number of iterations is determined based on expert experience and system history. The termination condition in this paper takes into account the time cost and decision results and has more application value in practical scenarios.

#### 3.2.5. Migration Operator Design

This paper adopts the idea of a parallel genetic algorithm and introduces the migration operator to realize the migration of individuals in each sub-population. The migration operator is mainly used to control the migration of individuals among sub-populations, as shown in the equation

(2) $$\begin{array}{c} \text{Transport}=\left\{\text{population migration rate,migration cycle, migration strategy,migration topology}\right\}. \end{array}$$

In formula (2), population migration rate refers to the proportion of new individuals among sub-populations in the atomic population. The migration period refers to the time interval of individual migration between sub-populations; Migration strategy refers to the individual migration strategy among sub-populations, which generally includes how to select the individuals who migrate outward, the replacement strategy after the population receives the individuals, and the retention strategy of the individuals who migrate out. Migration topology refers to the migration path of individuals between populations.

**Figure 3:**
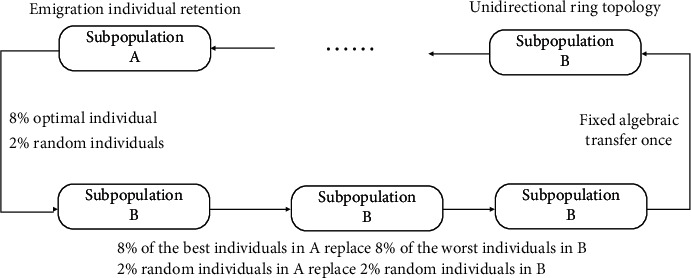
Migration operator design.

The genetic operator setting in this paper is shown in Figure 3. After many experiments, to ensure the parallel efficiency, this paper sets the population migration rate to 10%. As for the migration strategy, the current main replacement methods include replacing the worst individual or random replacement. Selecting the best individual can accelerate the convergence efficiency of the algorithm. However, selecting random individual migration may obtain better results because it expands the diversity of the population after a period of time. In this paper, the above two methods are comprehensively considered, and the convergence efficiency of the algorithm is more important. At the same time to reduce the overhead, this paper chooses the idea of synchronous migration and migrates every *T* generation. The migration topology adopts one-way ring topology.

After introducing the migration operator, the PSOGA algorithm will be divided into several sub-populations. The multi-threaded technology is used to search the scheme space in parallel, and the migration operator is used to realize individual migration and information interaction. Finally, the optimal solution set is obtained, and the parallel search is realized to improve the search efficiency.

### 3.3. Multi Index Sorting Method Based on ELECTRE

In view of the large battlefield space, many uncertain factors, and strong variability of the C3I system strategy, it is necessary to improve the decision efficiency and ensure the scientificity of the decision in wartime. Therefore, based on the ELECTRE method, this paper quantifies the commander's preference, adjustment cost, adjustment time, and other evaluation indicators related to the C3I system, and then combines the concepts of harmony and disharmony to set up two test methods to achieve the evaluation and ranking of the frontier adaptive strategy set.

In terms of the harmony test, this paper quantifies the evaluation indicators of user preference and adjustment cost. On this basis, it compares the quality of Frontier adjustment strategies to solve the problem that the adjustment strategies are not unique due to the different results of different objective functions and further optimize the selection of the best strategy.

Among them, the user preference in the battlefield environment mainly refers to the command preference, which is the weight value set for different objective functions, usually in the process of strategy generation. On this basis, the total score of scheme *i* after integrating all objective functions is calculated by weighting, that is, the user preference score Score_Preference_*i*_.

The adjustment cost represents the evolutionary reconfiguration cost for the adaptive decision mechanism to execute a specific adjustment scheme. It includes resource consumption “costresources,” time consumption “costtime,” and scope impact “costscope.” First, the sum of the costs is calculated according to expert experience or actual tests to represent the adjustment cost “costi” of the adjustment scheme *i*; Then, the adjustment cost score Score_Costi is calculated by comprehensively considering the ratio of the adjustment cost of strategy *i* to the adjustment cost of all strategies. The higher the adjustment cost score is, the more advantages this strategy has in all front-set adjustment strategies.

On the basis of the above two evaluation indexes, combined with the decision experience of the adaptive system, this paper sets the weight value for different evaluation indexes to calculate the total score of the strategy. Among them, this paper considers the adaptive decision requirements of the C3I system, and through consulting the experience of experts, considers that the influence of user preference on the decision results should be greater than the adjustment cost in the strategy selection process in the battlefield environment. Therefore, this paper sets the proportion of user preference to 0.7 and adjusts the cost proportion to 0.3. As shown in formula (3), the final total score_*i*_ can be calculated.

(3) $$\begin{array}{c} {\text{Score}}_{i}=0.7\times \text{Score}\_{\text{Preference}}_{i}+0.3\times \text{Score}\_{\text{Cost}}_{i}. \end{array}$$

In terms of the disharmony test, this paper sets the threshold that users can accept for the evaluation index and objective function, including user preference, adjustment cost, and objective function results. By taking the threshold value as the basis to measure whether the adjustment strategy is inharmonious or not, all the strategies whose scores are within the reasonable threshold value are screened, and further through the harmony test method, the best strategy is selected, which can meet the user preferences and effectively improve the rationality and applicability of the adjustment strategy.

Based on the above two parts of the strategy evaluation method, this paper establishes the main process of the multi-index ranking method, as shown in Algorithm 2. This method first obtains the state information of the variable nodes, calculates the total score of each adjustment strategy, and then selects the best adjustment strategy through the harmony test and disharmony test.

Therefore, the multi-index ranking method proposed in this paper can adapt to the more complex battlefield environment. For the adaptive decision problem of multi-objective function, it comprehensively analyzes the impact of various influencing factors on the adjustment strategy and quantitatively scores different adjustment strategies to select the only strategy. This method combines the user's command and control preference and uses expert experience to deal with the actual battlefield command and control needs. It can effectively make up for the limitations of manual participation and is conducive to improving the effectiveness and rationality of adaptive decision results.

## 4. Experimental Verification and Analysis

### 4.1. Experiments Design

To test the significance of the method, this paper attempts to design three experiments to verify it.

The search subsystem designed in this paper contains the composition information, equipment information, and environment information of a region. The system includes component information subject service, equipment information subject service, and environment subject service. Each type of service provides three functions: video search, image search, and basic information search. After obtaining relevant information, it will be displayed to the commander through analysis and sorting. In terms of system deployment, the system adopts multi-node distributed deployment. There are several services on each node, and each service has several instances according to requirements.

The search subsystem designed in this experiment consists of 13 nodes, each of which contains several data processing instances and perceptual monitoring instances. There are seven information maintenance nodes, numbered node1–node7. There are three information communication nodes, numbered node8 to node10. There are three information analysis nodes numbered node11–node13. The specific node configuration information is shown in Table 1. Each node contains eight basic service instances, which are respectively data retrieval service instances in charge of video search, image search and basic information search, data processing service instances in charge of composition information, equipment information, and environment information, and two perceptual monitoring instances in charge of perceptual information change adjustment.

A large-scale scenario is designed and simulated for damage test, and 4700 virtual nodes are simulated. Each node contains a sensing monitoring service and a data processing service. The experimental environment is shown in Table 2. There are 15 computers of this type, of which each computer establishes 2 virtual machine nodes, forming a cluster of 30 computing nodes under the microservice platform, and created 4700 virtual nodes through kubernet management tool.

### 4.2. Method Test

#### 4.2.1. Test of Adaptive Decision Method for Command and Control Field

In the damage replacement scenario, first, the service instance redeployment process needs to be mapped into a multi-objective optimization process according to the node information, environmental characteristics, and other factors. The damaged instances are redeployed to the selected nodes according to the results. Due to the different performance of each node, the redeployed services may cause the problem of node overload, Therefore, it is necessary to adjust the load of the deployed instance to make the system reach a better state. The specific experimental process and results are analyzed as follows:

*(1) Mapping the adaptive decision process*. The underlying environment information of the node itself, the interaction between nodes, and the domain characteristics of the military system. It is necessary to comprehensively consider the above factors to select replacement nodes and establish target functions to search for the optimal replaceable nodes. The specific indicators considered are as follows:

- The node's underlying environment information, that is, the node's CPU, memory, disk usage, availability, and total capacity. This information is necessary to ensure that the node can perform tasks normally.
- Communication capability between nodes. For the military information system, the complexity of its operating environment and the requirements of high real-time make the nodes inside the system need to interact frequently to ensure the normal operation of the system. Therefore, the communication ability between nodes is particularly important for the normal task execution of nodes.
- Reliability and security of nodes. Due to the particularity of military information systems in the military field, its data and nodes need a high degree of confidentiality, and security mechanism is also an indispensable consideration.

In view of the above three factors, this paper establishes five objective functions of CPU, memory, disk, network, and security in the search process of damage replacement, as follows:

(4) $$\begin{array}{c} x_{i}.\text{CpuAbility}=x_{i}.{\text{CPU}}_{\text{type}}\times \left(\frac{x_{i}.{\text{CPU}}_{\text{frequency}}-\text{min}\left({\text{CPU}}_{\text{frequency}}\right)}{\text{max}\left({\text{CPU}}_{\text{frequency}}\right)-\text{min}\left({\text{CPU}}_{\text{frequency}}\right)}+\left(1-x_{i}.{\text{CPU}}_{\text{used}}\right)\right). \end{array}$$

As shown in formula (4), *x*_*i*_.CpuAbility indicates the CPU capacity of node *x*_*i*_, where, *x*_*i*_.CPU_type_ indicates the CPU type of node *x*_*i*_, *x*_*i*_.CPU_frequency_ indicates the CPU processing frequency of node *x*_*i*_, *x*_*i*_.CPU_used_ indicates the CPU utilization of node *x*_*i*_.

(5) $$\begin{array}{c} x_{i}.\text{MemoryAbility}=x_{i}.{\text{Weight}}_{\text{Type}}\\ \times \left(\frac{x_{i}.{\text{Memory}}_{\text{speed}}-\text{min}\left({\text{Memory}}_{\text{speed}}\right)}{\text{max}\left({\text{Memory}}_{\text{speed}}\right)-\text{min}\left({\text{Memory}}_{\text{speed}}\right)}+\frac{1-x_{i}.{\text{Memory}}_{\text{used}}}{\text{max}\left({1-\text{Memory}}_{\text{used}}\right)-\text{min}\left({\text{Memory}}_{\text{used}}\right)}\right). \end{array}$$

As shown in formula (5), *x*_*i*_.MemoryAbility indicates the memory capacity of node *x*_*i*_. where, *x*_*i*_.Weight_Type_ indicates the memory type weight of node *x*_*i*_, *x*_*i*_.Memory_speed_ indicates the memory read/write speed of node *x*_*i*_, and *x*_*i*_.Memory_used_ indicates the memory utilization of node *x*_*i*_.

(6) $$\begin{array}{c} x_{i}.\text{DiskAbility}=x_{i}.{\text{Weight}}_{\text{Type}}\times \left(\frac{\left(x_{i}.\;{\text{Disk}}_{\text{speed}}\right)}{\left(\begin{array}{c} \text{max}\left({\text{Disk}}_{\text{speed}}\right)-\left(\text{min}\left({\text{Disk}}_{\text{speed}}\right)\right)+\\ \left(1-x_{i}.\;Di\;sk_{\text{used}}\right)-\text{min}\left(1-{\text{Disk}}_{\text{used}}\right)/\text{max}\left(1-{\text{Disk}}_{\text{used}}\right)-\text{min}\left(1-{\text{Disk}}_{\text{used}}\right)+\\ x_{i}.\;{\text{Disk}}_{\text{size}}-\text{min}\left({\text{Disk}}_{\text{size}}\right)/\max\left({\text{Disk}}_{\text{size}}\right)-\text{min}\left({\text{Disk}}_{\text{size}}\right)\times \varepsilon \end{array}\right)}\right). \end{array}$$

As shown in formula (6) *x*_*i*_.DiskAbility indicates the disk capability of node *x*_*i*_ . where, *x*_*i*_.Weight_Type_ indicates the disk type weight of node *x*_*i*_, *x*_*i*_.Disk_speed_ indicates the average disk read/write speed of node *x*_*i*_, *x*_*i*_.Disk_used_ indicates the disk utilization of node *x*_*i*_, *x*_*i*_.Disk_size_ indicates the disk size of node *x*_*i*_.

*(2) Destroy node instance redeployment decision*. To simulate the damage replacement scenario, this paper closes node node7 in the search subsystem to simulate the damage to the node. The node deploys eight instances in total, including three data processing service instances with instance numbers of I0, I1 and I2, three data retrieval instances with instance numbers of I3, I4, and i5, and two perceptual monitoring service instances with instance numbers of I6 and I7.

After the node is damaged, the sensing mechanism cannot obtain the information about the node, judge that the node is damaged, and issue the “damage replacement” event. The system receives the “damage and replacement” event and relevant information to trigger the decision behavior. At this timeto ensure the real-time performance of the search results of the search subsystem, the adaptive decision needs to redeploy the service instances on the damaged nodes to other nodes. According to the status of other nodes and taking into account the overall resource utilization of the nodes, the adaptive decision finds deployment nodes for the service instances on the damaged nodes in turn and calculates the scores of each node in the five dimensions of CPU, memory, disk, network, and security according to the objective function. The calculated score segments are shown in Table 3.

Through calculation, the scores of instance redeployment in each node are obtained. Since I0, I1, and I2 are data processing service instances that require more CPU and memory, it is decided to deploy them on node12 node. I3, I4, and i5 are data retrieval services with high memory and disk requirements, so it is decided to deploy them on node13 node. I6 and I7 are sensing monitoring service instances with high network and security requirements, so it is decided to deploy them on node9 node.

*(3) Deployment node load balancing adjustment decision*. Because the performance of computing nodes is different, the types and number of instances that can be deployed are different. Considering the limited load capacity of nodes, if the sensing mechanism monitors that the current system has a heavy load trend, the adaptive decision will trigger the load balancing to re-plan the deployment scheme.

Before load balancing adjustment, it is necessary to consider the data related to load balancing, such as node utilization and service operation efficiency, and establish an objective function to search for the optimal load adjustment scheme. The specific objective function is established as follows.

Node utilization score *S*_use_ comprehensively considers the utilization score of each resource of each node, as shown in

(7) $$\begin{array}{c} S_{\text{use}}=\sum_{i=1}^{n}\left(\alpha S_{{\text{cpu}}_{i}}+\beta S_{{\text{mem}}_{i}}+\gamma S_{{\text{disk}}_{i}}\right). \end{array}$$

The utilization score of a single node includes the CPU utilization score *S*_cpu_*i*__, memory utilization score *S*_mem_*i*__,and disk utilization score *S*_disk_*i*__, *α*, *β*, *γ*, *α*, *β*, *γ* are the three utilization weights respectively. The calculation method of each resource utilization is the ratio of the resources allocated to the container to the total resources.

Service operation efficiency *S*_cap_ is divided into two aspects: one is the operation efficiency score of a single service instance *S*_cap_*i*__, and the other is the efficiency score of communication between service instances *S*_*b*_cap_*i*__, as shown in

(8) $$\begin{array}{c} S_{\text{cap}}=\sum_{i=1}^{n}S_{{\text{cap}}_{i}}+\sum_{i=1}^{m}\omega_{i}S_{b\_{\text{cap}}_{i}}, \end{array}$$

where, *n* represents the number of service instances and *m* represents the total number of communication relationships between service instances. The operation efficiency score of a single service *S*_cap_*i*__ considers six aspects in total, including CPU quota, memory type, disk type, network failure rate, security level, and instrument security (IMTTF) indicators. The score of each aspect is calculated by the ratio of the actual information of the node to the expected value of the instance (the expected configuration information table of the node). Efficiency score of communication between service instances *S*_*b*_cap_*i*__ is calculated by traversing the communication relationship between known service instances. If both sides of the communication relationship are deployed on the same node, the score is 1; otherwise, the score is 0, and the weight *ω*_*i*_ is the strength of this communication relationship.

Then, the adaptive decision method calculates the optimal deployment strategy of the search subsystem on the remaining 12 nodes according to the objective function. If the 12 nodes cannot meet the resource requirements of the search subsystem, for the time being, the system will reduce the number of instances of some secondary services or even close some secondary services according to the selection to ensure the progress of the main search tasks. Finally, a set of policies suitable for adjusting the deployment is generated. Through the adaptive decision method, the optimal strategy adjusted within six nodes is obtained, as shown in Table 4.

*(4) Load balancing strategy selection based on post optimization theory*. When the adaptive mechanism executes the adjustment strategy, it must adjust the system structure and behavior according to the unique strategy scheme. Therefore, a complete adaptive decision method also needs to evaluate and sort according to the decision needs and selection indicators in different decision environments, combined with operational preferences, resource situations, and other indicators, based on the frontier strategy set generated by the above methods, to generate the optimal and unique adjustment strategy. The specific evaluation calculation method is as follows.

User preference Score_Preference_*i*_ is mainly used to calculate the comprehensive score of scheme I in all objective functions, that is, the user preference score, by weighting the weights set by the commander for different objective functions, as shown in formula (9), where, Preference_*i*_ is the user preference weight and Score_*i*_ is the score of different objective functions.

(9) $$\begin{array}{c} \text{Score}\_{\text{Preference}}_{i}=\sum_{i}^{n}\left({\text{Preference}}_{i}\times {\text{Score}}_{i}\right). \end{array}$$

Adjust Score_Cost_*i*_ calculates the ratio of the adjustment cost of strategy *i* to the adjustment time cost of all strategies, as shown in formula (10). Cost_*i*_ is the adjustment time cost of a specific policy and ∑_*i*=1_^*n*^Cost_*i*_ is the sum of the adjustment time costs of all policies.

(10) $$\begin{array}{c} {\text{Score\_Cost}}_{i}=\frac{1}{2}\left(1-\frac{{\text{Cost}}_{i}}{\sum_{i=1}^{n}{\text{Cost}}_{i}}\right). \end{array}$$

The total score of the deployment strategy Score_*i*_ comprehensively considers the time adjustment cost and user preference score, and the specific calculation is shown in

(11) $$\begin{array}{c} {\text{Score\_Cost}}_{i}=\frac{1}{2}\left(1-\frac{{\text{Cost}}_{i}}{\sum_{i=1}^{n}{\text{Cost}}_{i}}\right). \end{array}$$

Taking this scenario verification experiment as an example, in the above strategy set, assuming that the node resources are limited, more consideration should be given to the node resource utilization. The score of strategy 1 is slightly higher than that of strategy 2, but if you want to better provide services, strategy 2 is significantly better than strategy 1. Therefore, considering that the above two strategies have little difference in resource utilization indicators, this paper sets the user preference weight of service operation efficiency to 0.7. The user preference weight of node utilization was set to 0.3. The user preference scores of different strategies was calculated. Then, the adjustment behavior time cost of each service instance was set to 1, and policy 1 adjusts 5 service instances, Cost_1_ to 5; Strategy 2 adjusts 5 service instances, Cost_2_ is 5. According to formula (11), the cost score of strategy 1 is Score_Cost_1_=0.3. The cost score of strategy 2 is Score_Cost_2_=0.2. Finally, the total score of the final strategy is calculated according to formula (12), as shown in Table 5.

(12) $$\begin{array}{c} {\text{Score}}_{i}=0.7\times \text{Score}\_{\text{Preference}}_{i}+0.3\times \text{Score}\_{\text{Cost}}_{i}. \end{array}$$

Therefore, according to the total score, this paper selects strategy 2 with better overall service quality performance as the strategy for system deployment and adjustment.

**Figure 4:**
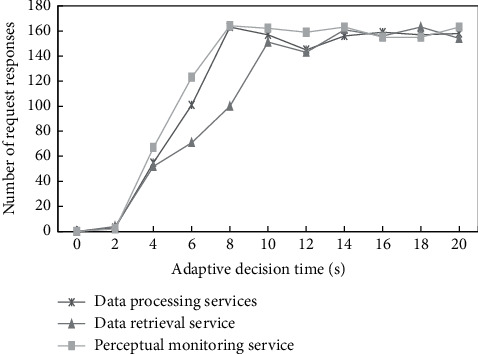
Graph of the number of requests and responses per second.

To better illustrate that this method can ensure the normal and stable operation of the system, this experiment tests the number of requests and responses per second. By sending a large number of requests to three types of services in the damaged node, the number of requests and responses per second is observed. If the number of requests per second remains within the normal range, the system is in a stable operation state.  Figure 4 illustrates the test result.

From the test results, it can be seen that the number of request responses only remains in single digits during the beginning of the adaptive decision process. This is because the algorithm requires a certain execution time, and it takes a certain time to redeploy the instances on the damaged node to other nodes and make adjustments. At the same time, the number of requests and responses has recovered to the normal range and tends to be stable between 7 and 8 s. This is because the instances on the damaged node have been adjusted and deployed, and the system has returned to a stable running state. This time interval has been maintained within 10 s, which can meet the needs of the command and control field. Therefore, the method can effectively carry out adaptive adjustment.

**Figure 5:**
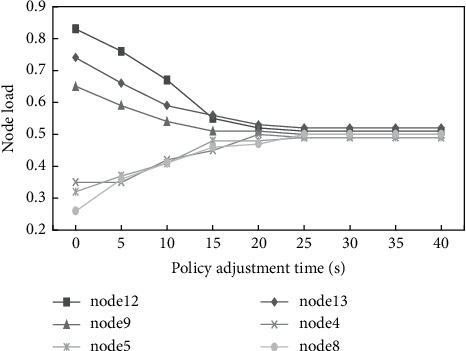
Node load variation diagram.

At the same time, to verify that the final policy generated by this method during policy adjustment is effective, this paper monitors the node load of the adjusted nodes, mainly monitoring the node load of node2, node4, node8, node9, node12 and node13. The experimental results are shown in Figure 5.

From the test results, it can be seen that at the beginning of the policy adjustment, the damaged nodes were centrally deployed on node9, node12, and node13 nodes, resulting in a load of these three nodes being too high and deviating from the normal load range, while the load of node4, node5, and node8 nodes being too low was not fully utilized. From the figure, we can see that the load of the nodes deviating from the normal load range gradually became reasonable and stabilized after the implementation of the adjustment policy. This proves that the strategy generated by this method can effectively adjust the system and make the system run smoothly.

From the above experiments, it can be seen that the adaptive decision method proposed in this paper can realize the transformation from adaptive decision problem to an optimization problem in the scenario of damage replacement in the command and control field. At the same time, it can realize the trade-off decision of strategies for a variety of factors, and finally produce a unique strategy through the post optimization theory.

#### 4.2.2. Performance and Robustness Test

To verify the performance and robustness of this method, this paper designs the test experiment of algorithm response time in large-scale environment and the effect of generation strategy under different damage degrees. The specific experiments are as follows.

**Figure 6:**
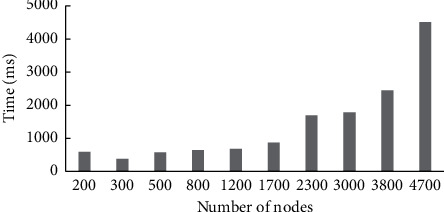
Number of nodes and response time of adaptive decision.

**Figure 7:**
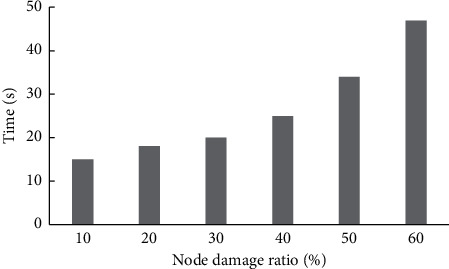
Time varies with nodes.

1. Algorithm performance test in large-scale environment.
2. To verify that the method is still real-time in large-scale scenarios, this paper tests the response time of the adaptive decision algorithm, and observes the response time by adjusting the number of nodes. The test starts from 200 nodes, and increases 100, 200, 300, 400, 500, 600, 700, 800, 900 nodes to 4,700 nodes in turn. Observe the change in the response time of the algorithm, conduct 10 experiments for each scale, and take the average value as the experimental result. The number of nodes and adaptive decision response time are shown in Figure 6.
3. It can be seen from the test results that the response time of the proposed adaptive decision method is always within an acceptable range with the rapid increase of computing nodes. Among them, the algorithm response time is more than 2 s under 3800 optional nodes and less than 1 s under 1700 analog nodes. This shows that although the computing scale in the command and control field becomes larger, this method can still meet the high real-time requirements of military systems.
4. Method robustness test in an extreme environment.
5. Considering that in the extreme environment, the node damage ratio will reach a high proportion, to verify the robustness in this extreme environment, this paper observes the change of the system node damage adjustment time by adjusting the node damage ratio. In this experiment, the test range of damage ratio of test nodes is determined as 10%–60%, and the damage ratio of each node is increased by 10%. Ten experiments were conducted for different node damage ratios, and the average value of each time was taken as the node damage adjustment time. The node damage ratio and adjustment time are shown in Figure 7. The horizontal axis represents the node damage ratio, and the vertical axis represents the node damage adjustment time. The time unit is s.

It can be seen from the test results that when the node damage ratio is 10%–60%, the system operates normally, the node damage adjustment speed is fast, and the adjustment time is not more than 1 min, which can ensure the continuous and reliable operation of the military system. Therefore, the proposed method can produce effective adjustment strategies to ensure the smooth operation of the system in extreme environments.

#### 4.2.3. Comparative Experimental Tests

This paper prepares two types of comparative tests of strategy efficiency and strategy effect, simulates the application of this method and other related methods in the actual scenario, and aims to test and evaluate the effectiveness, advantages, and disadvantages of various methods. The specific experiments are as follows.

**Figure 8:**
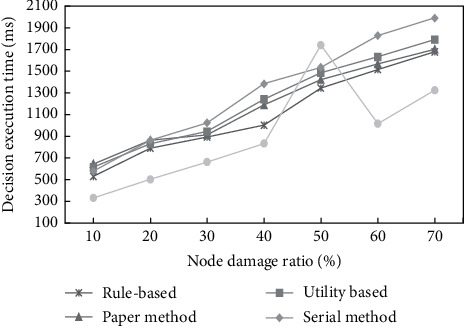
Diagram of time-consuming decision process changing with the proportion of damaged nodes.

**Figure 9:**
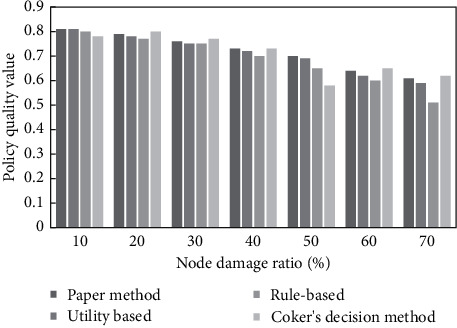
Variation of strategy quality value with the proportion of damaged nodes.

1. Decision efficiency test.
2. In the efficiency test of the decision method, this paper takes the time-consuming decision process (index 1) as the measurement standard, tests and evaluates the efficiency of the serial version of the adaptive decision method and the parallel search adaptive decision method, the composite adaptive decision method [24], the rule-based adaptive decision method [25] and the utility-based adaptive method [26] in the damage and replacement scenario. The parallel version refers to using parallel optimization mechanism to execute this method in a multi-threaded manner by dividing the population. The method of using two versions is to verify the effectiveness of the parallel effective mechanism. First, a comparative test is conducted with reference to the configuration environment of question 1 above. Then, 20 tests are conducted for different node damage ratios, and the average value of each test is taken as the time-consuming of the decision process. The node damage ratio and decision process time are shown in Figure 8. The abscissa represents the node damage ratio, and the vertical axis represents the node damage adjustment time. The time unit is ms.
3. From the comparison test results of search-based methods, it can be seen that the self-adaptive decision method proposed in this paper and the composite decision method proposed by [27], with the increase in damage ratio, the time-consuming of the decision process is also increasing, but neither of them is more than 2 s. It is obvious that the decision process of parallel method is generally less time-consuming than that of the serial method. In most cases, the time consumption of [28] the decision method is generally lower than that of serial and parallel methods. This is because their decision method, as a static decision method, is shorter than that of the dynamic decision method. However, when the number of damaged nodes is 50%, the decision process time of the static decision method increases significantly, which is higher than that of the serial and parallel methods. This is because the rule base of the static offline method has certain limitations, and all strategies and event mappings need to be set in advance. When there is no adjustment strategy in the case of 50% damage, it is necessary to search and traverse the complete feasible solution space, After the search fails, select the adjustment strategy when 40% damage is similar to the situation.
4. Compared with other methods in the field of command and control, the test results show that with the increase of the damage ratio of nodes, the decision execution time of the adaptive decision method based on the parallel search proposed in this paper will increase, and its decision execution time in the whole process is basically higher than that of the rule-based adaptive decision method, and there is little difference between the decision execution time of the rule-based adaptive decision method and that of the utility-based adaptive decision method. The decision execution time of the method proposed in this paper is basically higher than that of the rule-based adaptive decision method when the damage ratio of nodes is 10%–30%. This is because the rules judged by the rule-based adaptive decision method are often carried out in a static way, and the decision time is less. When the damage ratio of nodes is higher than 40%, the damage ratio of the whole system is too high and the number of nodes is reduced. In order to meet the system requirements, each node must increase the number of its own threads, resulting in a sudden increase in the line graph of its decision execution time, but it is still lower than the utility-based adaptive decision method.
5. Strategy effect test.
6. In the effectiveness test of decision methods, this paper takes the strategy quality value index (2) as the measurement standard to measure and evaluate the advantages and disadvantages of the above four decision methods, except the serial method. Firstly, based on the time-consuming test environment for the above decision process, this paper conducts 20 tests for different node damage ratios, calculates the strategy quality values of different strategies each time, and takes the average value. The time consumption of node damage ratio and policy quality is shown in Figure 9, where the abscissa represents the node damage ratio and the vertical axis represents the policy quality.
7. From the comparison test results of the search-based method, it can be seen that the quality values of the adaptive decision method and the static method proposed in this paper have similar strategic effects under different damage ratios in the damage replacement scenario, and there is a certain gap with the quality values of the ideal points, and they continue to decrease with the increase of the damage ratio. In the case of 50% damage, the mass value of the static method decreases significantly and is lower than that of 40% and 60% damage. The reason is that in the case of 50% damage, the static method fails to search the feasible solution space, and then selects the adjustment method of 40% damage. However, due to the increase in the damage proportion, the same adjustment scheme is not applicable to the case of 50% damage, and the strategy effect is lower than that of 40% damage. Therefore, in practical application, the adaptive decision method based on parallel search optimization can generate more stable and reliable strategies at the expense of a certain time-consuming decision process, while the static method may achieve faster results in the process of adaptive decision. However, there are certain probability rule matching failures, which seriously affect the software operation and do not meet the needs of actual military application scenarios.
8. Compared with other methods in the command and control field, the test results show that the policy quality values of the three methods decrease with the increase of the damage ratio of nodes. This shows that the damage ratio of nodes has a direct impact on the policy quality because with the increase in the damage ratio of nodes, the process of policy optimization becomes more and more complex. The search-based adaptive decision method proposed in this paper is superior to the rule-based and utility-based methods under different damage ratios. When the damage ratio is less than or equal to 40%, the difference between the policy quality values of the three methods is small. When the damage ratio ≥50%, the number of rule-based policy quality is significantly lower than that of the other two methods. This is because the rule-based method cannot dynamically generate rules according to the current scenario, and the quality of its policy will be reduced. When the policy quality value is close, the method in this paper is less time-consuming than the utility-based method. This is because the utility-based method needs five calculations each time in the implementation process, so it is time-consuming. According to the above comparison, it can be concluded that the policy quality and policy execution time of this method are significantly better than the other two methods, which proves the superiority of this method in policy quality and time cost.

## 5. Conclusion

In this paper, an adaptive decision-making method for command and control systems based on parallel search optimization is proposed. By transforming the adaptive decision-making problem into a search optimization problem, a multi-objective optimization algorithm is designed to meet the needs of online decision-making and decision-making trade-off of command and control systems. At the same time, a strategy selection method based on post optimization theory is designed to improve the decision-making efficiency, which can select the most applicable strategy at present It includes the following points: first, adaptive decision problem modeling. In this paper, the characteristics of adaptive decision problems are analyzed. The adaptive strategy, strategy space, objective function, fitness function, and constraint function of this kind of problems are defined. The adaption decision question is modeled as retrieval optimization problem. The adaptive decision modeling method designed in this paper can quickly model a unique adaptive decision problem model according to the characteristics of the C3I system, transform the adaption decision question into retrieval optimization problem, dynamically generate a strategy space according to the changes of the C3I system environment. Second, parallel-based multi-objective optimization algorithm. In this paper, a parallel adaptive decision method based on particle swarm optimization and genetic algorithm (PSOGA) is established by combining the theories of parallel genetic algorithm and particle swarm optimization, and the parallel design of the algorithm ensures that the algorithm can quickly generate adjustment strategies. This method can not only generate the optimal policy online for multiple environmental changes in the complex operating environment of the C3I system but also avoid the policy conflict caused by considering only a single change. In addition, the parallel design of the algorithm is realized by means of population cutting and introducing a migration operator, which improves the decision efficiency. Third, the strategy selection method is based on post optimization theory. Based on the post optimization theory, aiming at the different characteristics of different decision problems in decision preferences, timeliness constraints, and so on, this paper establishes a multi-index ranking method based on the ELECTRE, which can select the most suitable strategy at present so as to guide the C3I system to realize adaptive adjustment.

## Figures and Tables

**Algorithm 1:**
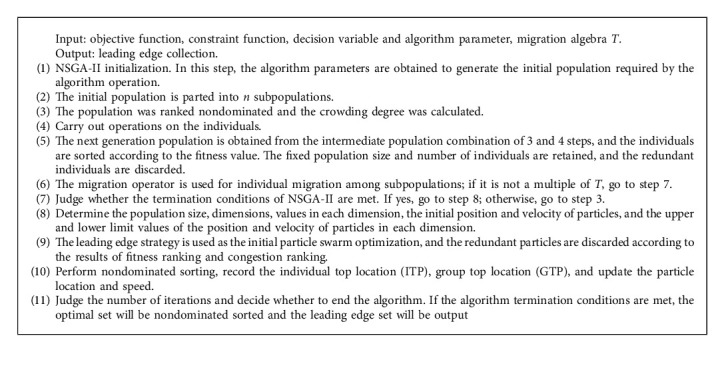
PSOGA algorithm flow.

**Algorithm 2:**
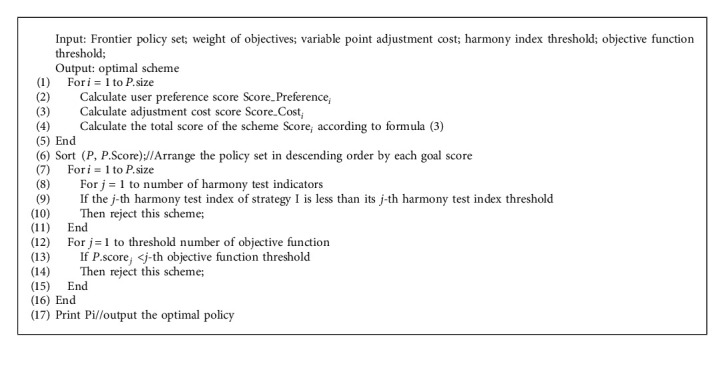
Multi-index ranking method.

**Table 1:** Node configuration information table.

Node category	CPU	Memory	Hard disk	Network
Information maintenance nodes (7 in total)	Model: Intel i5; frequency: 2.5 GHz; utilization ratio: 0.80	Type: DDR2; speed: 19 gb/s; utilization ratio: 0.89	Type: raid0; speed: 0.12 gb/s; utilization ratio: 0.8; capacity: 0.9 TB	Packet loss rate: 0 42; utilization ratio: 0.62; inflow flow: 99321024B; outflow flow: 14423570B

Information communication nodes (3 in total)	Model: Intel i5; frequency: 3.5 GHz; utilization ratio: 0.70	Type: DDR3; speed: 20 gb/s; utilization ratio: 0.69	Type: raid0; speed: 0.2 gb/s; utilization ratio: 0.4; capacity: 1 TB	Packet loss rate: 0 12; utilization ratio: 0.12; inflow flow: 99348024B; outflow flow: 14183570B

Information analysis nodes (3 in total)	Model: Intel i5; frequency: 3.5 GHz; utilization ratio: 0.70	Type: DDR3; speed: 30 gb/s; utilization ratio: 0.70	Type: raid0; speed: 0.22 gb/s; utilization ratio: 0.4; capacity: 1 TB	Packet loss rate: 0 12; utilization ratio: 0.12; inflow flow: 99358024B; outflow flow: 15153570B

**Table 2:** Experimental computer information.

Environment	Parameter	Value
Physical machine environment	Computer model	OptiPlex 7050
	Processor	Intel core i7 3.5 GHz×4
	Memory	16 GB
	Hard disk	2 TB

Virtual machine environment	Virtual machine software	VMware® workstation 15 pro
	Memory	3 GB
	Number of processor cores	2
	Hard disk	50 GB

**Table 3:** Example of service scoring segments at each node.

Instance name	Deployment node	CPU score	Memory score	Disk score	Network score	Safety score
I0	1	0.60	6.15	6.09	6.03	16500
	2	0.70	6.24	6.17	6.11	24200
	3	0.62	5.88	5.82	5.76	18500
	…	…	…	…	…	…
	12	0.73	6.34	6.27	6.21	16600
	13	0.64	6.25	6.18	6.12	36100

I1	1	0.64	6.28	6.21	6.15	16500
	2	0.72	6.30	6.23	6.17	24200
	3	0.64	6.07	6.01	5.95	18500
	…	…	…	…	…	…
	12	0.78	6.57	6.51	6.44	16600
	13	0.72	6.34	6.27	6.21	36100

…	…	…	…	…	…	…

I7	1	0.53	6.18	6.12	6.05	16500
	2	0.64	6.29	6.22	6.16	24200
	3	0.60	5.86	5.80	5.74	18500
	…	…	…	…	…	…
	12	0.76	6.35	6.28	6.22	16600
	13	0.73	6.26	6.19	6.13	36100

**Table 4:** Better instance adjustment strategy.

Deployment strategy	Node2	Node4	Node8	Node9	Node12	Node13	Node utilization score	Service operation efficiency score
Strategy 1	INSTANCE2	INSTANCE1	INSTANCE3, INSTANCE5	INSTANCE6	INSTANCE0, INSTANCE7	INSTANCE4	1.3200	0.8768

Strategy 2	INSTANCE3	INSTANCE1	INSTANCE2, INSTANCE5	INSTANCE6	INSTANCE0, INSTANCE7	INSTANCE4	1.3150	0.9607

**Table 5:** Strategy evaluation calculation table.

Deployment strategy	Node utilization score	Service operation efficiency score	User preference score	Adjustment overhead	Cost score	Total score
Strategy 1	1.3200	0.8768	1.00981	6	0.24	0.781117
Strategy 2	1.3150	0.9607	1.067062	6	0.24	0.821193

## Data Availability

The data used to support the findings of this study are available from the corresponding author upon request.
